# How to reprogram human fibroblasts to neurons

**DOI:** 10.1186/s13578-020-00476-2

**Published:** 2020-10-12

**Authors:** Ziran Xu, Shengnan Su, Siyan Zhou, Wentao Yang, Xin Deng, Yingying Sun, Lisha Li, Yulin Li

**Affiliations:** 1 The Key Laboratory of Pathobiology, Ministry of Education, College of Basic Medical Sciences, Jilin University, Changchun, 130021 People’s Republic of China; 2 The Second Hospital of Jilin University, Jilin, Changchun, 130041 China; 3 Department of Stomatology, The First Hospital of Jilin University, Changchun, 130021 People’s Republic of China; 4 Norman Bethune College of Medicine, Jilin University, Changchun, 130021 People’s Republic of China

**Keywords:** Human fibroblasts, Reprogramming, Transcriptional factors, Small molecules, Physical factors, Neurons

## Abstract

Destruction and death of neurons can lead to neurodegenerative diseases. One possible way to treat neurodegenerative diseases and damage of the nervous system is replacing damaged and dead neurons by cell transplantation. If new neurons can replace the lost neurons, patients may be able to regain the lost functions of memory, motor, and so on. Therefore, acquiring neurons conveniently and efficiently is vital to treat neurological diseases. In recent years, studies on reprogramming human fibroblasts into neurons have emerged one after another, and this paper summarizes all these studies. Scientists find small molecules and transcription factors playing a crucial role in reprogramming and inducing neuron production. At the same time, both the physiological microenvironment in vivo and the physical and chemical factors in vitro play an essential role in the induction of neurons. Therefore, this paper summarized and analyzed these relevant factors. In addition, due to the unique advantages of physical factors in the process of reprogramming human fibroblasts into neurons, such as safe and minimally invasive, it has a more promising application prospect. Therefore, this paper also summarizes some successful physical mechanisms of utilizing fibroblasts to acquire neurons, which will provide new ideas for somatic cell reprogramming.

## Introduction

Three major steps happen in the human body during the development of CNS (central nervous system): the neurogenesis and mitosis of neural progenitor cells, migration to a particular position and differentiation of neurons under gene regulation [1]. Neural progenitor cells will be able to differentiate into neurons and neuroglial cells during human’s whole life cycle [2]. Neuroglial cells differentiated from neural progenitor cells provide microenvironment for the proliferation and growth of neurons, participating in neuronal signaling and immune defense [3–6]. Neurons can be divided into sensory neurons, motor neurons, and interneurons based on different functions [7–9]. Another way of classification shows that neuronal surface receptors are different, so the neurotransmitter signals they receive are different. Therefore, people can treat neurological diseases by aiming at different surface receptors of neurons. For example, the antagonist against the GABA_A_ receptor can improve spatial learning in rats, which is hoped to treat Alzheimer's disease [10]. In addition, there are experiments using neurochemical substrates to treat psychosis and neurological system diseases, including applying selective 5-tyrosine hydroxylase (5-HT) reuptake inhibitors to treat depression. Also, measuring extracellular histamine and acetylcholine has provided strategies to improve cognitive performance by, for example, blocking 5-HT_6_ or dopamine D3 receptors [11].

In addition to the above treatment of aiming at different surface receptors of neurons, people also pay attention to cell replacement therapy (CRT) for CNS diseases and applied this therapy to animal models. In recent years, people are starting to apply neurons to the treatment of disease models, studies of reprogramming human fibroblasts to neurons have emerged so rapidly, to gain the neurons from the most suitable way, we analyze three routes to obtain neurons in this paper.

The first route is to obtain induced pluripotent stem cells (iPSCs) by reprogramming human fibroblasts and then obtain neurons differentiated from iPSCs to treat nervous system. Cholinergic neurons induced by human mesenchymal stem cells (MSCs) were used to treat Alzheimer's disease and effectively improved the spatial learning and memory ability of AD rats [12]. In addition, human amniotic MSCs were pointed out the therapeutic characteristics in multiple sclerosis [13], and embryonic stem cells (ESCs) or iPSCs were applied to CRT, or direct lineage reprogramming in animal models of Parkinson's disease, these cells survived, activated, integrated, and provided functional recovery [14]. However, the main problems of using omnipotent cells as the starting material for CRT are their incomplete differentiation and their tendency to form tumors after transplantation [15].

The second route is to obtain neural stem cells (NSCs) by reprogramming human fibroblasts and then obtain neurons differentiated from NSCs to treat nervous system. The method used in the past to obtain neural stem cells from primary fetal brain tissue was ethically controversial and greatly exacerbated the likelihood of immune rejection and contamination in that cells from multiple fetuses were used in a single graft [16]. Therefore, the induction of NSCs reprogramming from human fibroblasts has great potential in CRT and models of nervous system diseases in vitro. This kind of NSCs has also been used in the treatment of disease models. Several key transcriptional factors (TFs) of neural progenitor cells were used to reprogram mouse and human fibroblasts to NSCs, which effectively ameliorated cognitive dysfunction of AD mice [17]. Neurons and astrocytes differentiated from NSCs were regulated by miRNA-146a to treat neurodevelopmental disorders [18], in addition, rats’ astrocytes were reprogrammed to NSCs which were used in the treatment of Neurodegenerative disease [6]. Considering the quantity of cells needed for CRT and the viability of transplanted cells, it is promising to select directionally differentiated NSCs for CRT.

In addition to the two routes mentioned above, the third route is to directly reprogram human fibroblasts to neurons. The direct reprogramming of human fibroblasts to neurons skips the multi-functional phase stage and the neurons have patient specificity, so the immune rejection reaction is reduced, and the ethical problems and oncogenicity brought by the iPSCs are also avoided. In this reprogramming process, some TFs and small molecules (SMs) play an important role. It opens up ideas for clinical application and has potential application value. For example, The combination of TFs BRN2, ASCL1, MYT1L, and NEUROD1 was used to reprogram human fibroblasts into neurons, which was the earliest research found in this paper and it opened up a new world for regenerative medicine [19]. Due to the experiment above, the feasibility of this treatment is proved. Ladewig. J et al. greatly increased the efficiency of reprogramming by introducing SMs, which may improve the efficiency of clinical application in the future [20]. At present, researchers can directly reprogram human fibroblasts into motor neurons, cholinergic neurons, dopaminergic neurons, and other types of neurons through TFs and/ or SMs, which makes the research on this way more diversified and promotes the progress of regenerative medicine. However, the introduction of ectopic genes (the TFs) is not completely controllable, which greatly limits the clinical application of this method. At the same time, the application of TFs also has some shortcomings, such as complex operation, long time consumption, low induction efficiency and so on, which weakens its application value; although SMs have many advantages, such as simple operation, easy control of the processing time, low experimental cost and the concentration and combination which can be adjusted artificially, the application of SMs also have a few disadvantages. The combination of TFs and SMs was used to reprogram, but the result was a mixture of different kinds of neurons [20]. And TFs were used to obtain pure cholinergic neurons, so we considered that during the reprogramming, TFs played a decisive role in the subtypes of neurons [21]. Except for that, Qin et al., Wan et al., and Hu et al. only used SMs to reprogram fibroblasts to neurons. However, due to the use of the excessive number of SMs and the unclear mechanism of part of the SMs, they haven’ t been used in vivo [8, 22, 23].

Therefore, the new methods have been explored to reprogram human fibroblasts directly to neurons, among which physical factors have their unique advantages. Directly applying physical factors to induce cells reprogramming can avoid the introduction of TFs and direct operation to genetic material. It not only plays a therapeutic effect but also safer. Physical factors can work in vivo to directly reprogram. For example, radio electric asymmetric conveyer (REAC) technique has been used in the treatment of Alzheimer’s patients, and it has been proved that there is no side effect while having a certain therapeutic effect [24]. It can also promote the reprogramming of human fibroblasts in vitro. By using physical factors, reprogramming human fibroblasts to NSCs or neural cells in vivo and in vitro has a great prospect in the treatment of nervous diseases.

## Different methods of reprogramming somatic cells to neural lineage

Currently, many methods can be used to obtain neural cells. To exclude immunological rejection, the patient's own cells should be given priority as the initial cells. Some scientists firstly get the patient's somatic cells or iPSCs derived from the somatic cells and then use SMs, reprogramming factors, microRNA and mRNA to down-regulate somatic genes and up-regulate nerve-related genes to reprogram somatic cells directly or indirectly into multiple neural cells including neural progenitor cells, NSCs, microglias, oligodendrocytes, astrocytes, and neurons. This method can be further applied from the laboratory to clinical research to test neurotoxic drugs in vitro to select drugs as well as treat some disease models such as Alzheimer's disease, amyotrophic lateral sclerosis, and Parkinson's disease [15]. Although colloid cells make up about half of the cells of the CNS [25], their main functions are auxiliary, supporting, nutrition, and so on while neurons performing the main function of the brain. In 2006, Yamanaka et al. used four factors to reprogram human fibroblasts to iPSCs [26]. Then the scientists asked whether they could bypass the iPSCs phase and reprogram human fibroblasts into neurons, which started with human fibroblasts and got neurons through direct or indirect reprogramming [27]. It can be divided into the following three ways.

## Reprogramming human iPSCs into neurons

Human fibroblasts can be reprogrammed into transient pluripotent cells firstly, from which neural precursor cells (NPCs) or iPSCs can be obtained and then lead to neurons [27]. Much time will be wasted by using conventional methods to make iPSCs differentiate into neurons. If iPSCs were induced into neurons with the mature neural network under certain growth conditions, the whole induction process took more than 95 days [28]. However, Canfield et al. first differentiated iPSCs into cell aggregates (a stable and extensible neural stem cell-like aggregation system which is derived by iPSCs), and then differentiated cell aggregates into neurons, which was more convenient and faster. The cell aggregates derived from iPSCs produced neurons populations in a relatively short period of 14 days [29]. In addition, more attention has been paid to the physical methods of promoting the differentiation of iPSCs into neurons. For example, by using tilapia collagen (COL) and adding cross-linking agents, a culture substrate was established which was a kind of gel with similar hardness to living brain tissue (150–1500 Pa). Exposure to gels with stiffness of approximately 1500 Pa during the early period of neural induction promoted the production of dorsal cortical neurons. This finding suggests that brain-stiffness-mimicking gel has the potential to determine the terminal neural subtypes. It proves that the gel simulating brain hardness has the potential to determine the subtypes of induced terminal neurons. The gel had the effect of hardness on the differentiation of iPSCs into neural lineages [30]. COL, hyaluronic acid and alginate with methacrylic anhydride were modified to photo crosslinked for graphed particles and then grafted with GRGDSP and Ln5-P4. After that, they self-assembled to integrate the microgel into three-dimensional scaffolds, by which they significantly improved the entrapment efficiency and viability of iPSCs and triggered the differentiation of iPSCs into neurons [31]. Except that, murine iPS-derived embryoid bodies were seeded on fibronectin or COL I-coated polyacrylamide gels of tunable stiffness in the presence of basal culture mediums, and used this soft matrix to culture iPSCs, achieving strong differentiation to neural lineages. To enhance the effect of neural differentiation, we could also deal iPSCs with a matrix composed of RA, NOGGIN and bFGF cytokines [32]. The differentiation of iPSCs into neurons has merged to its mature stage and compared with ESCs, iPSCs have certain advantages, however, iPSCs have some limitations, which limit their application in the clinic. For example, abnormal gene expression and epigenetic abnormal expression accumulated in iPSCs [33]. What is more, after iPSCs’ transplantation, there are potential risks of gene insertion mutagenesis and teratoma formation. These reasons limit the development and use of this technology [14]. Therefore, it is urgent to find new methods that can be used to differentiate human fibroblasts into transient pluripotent cells, which then can be differentiated into neurons. For example, during the induction of fibroblasts into iPSCs, Li X et al. fine-tuned the chemical formulation (VC6TFAE), replacing CHIR99021 with td116-2, so that the cells could be directly reprogrammed into functional neurons through the state of extra-embryonic endoderm-like state, thus bypassing the stage of NPCs. These neurons have neuron-specific expression profiles and form functional synapses in culture, which have the functional characteristics of neurons [34]. Besides, during the induction of fibroblasts into iPSCs, the cells were induced first to the embryoid state, then to the induced NPCs and finally into neurons to generate a functional and mature neural network [35]. In addition, a new induction system was proposed, in which miR-9/9 *, miR-124 and *BCL-XL* were used to overexpress *NGN2* to generate mature neurons, and it was proved that adding them to the *NGN2* expression system of iPSCs could enhance the neuronal maturation of differentiated cells. Notably, the resulting neurons showed increased calcium activity and synaptic formation. In addition, the microelectrode array analyses showed that the electrical network activity was very high [36]. Moreover, a cocktail containing MEK inhibitor PD0325901, GSK3β inhibitor CHIR99021, TGF-β/Activin/Nodal receptor inhibitor A-83–01, ROCK inhibitor HA-100 and human leukemia inhibitory factor, a medium with bFGF and N2B27 supplements and the human ESC medium mTeSR1 was used to successfully established a feeder-free reprogramming condition, greatly improved the episomal reprogramming efficiency, and succeeded in generating iPSCs, then the iPSCs were differentiated into motor neurons based on dual SMAD inhibition [37, 38].

## Reprogramming human fibroblasts into neurons

**Fig. 1:**
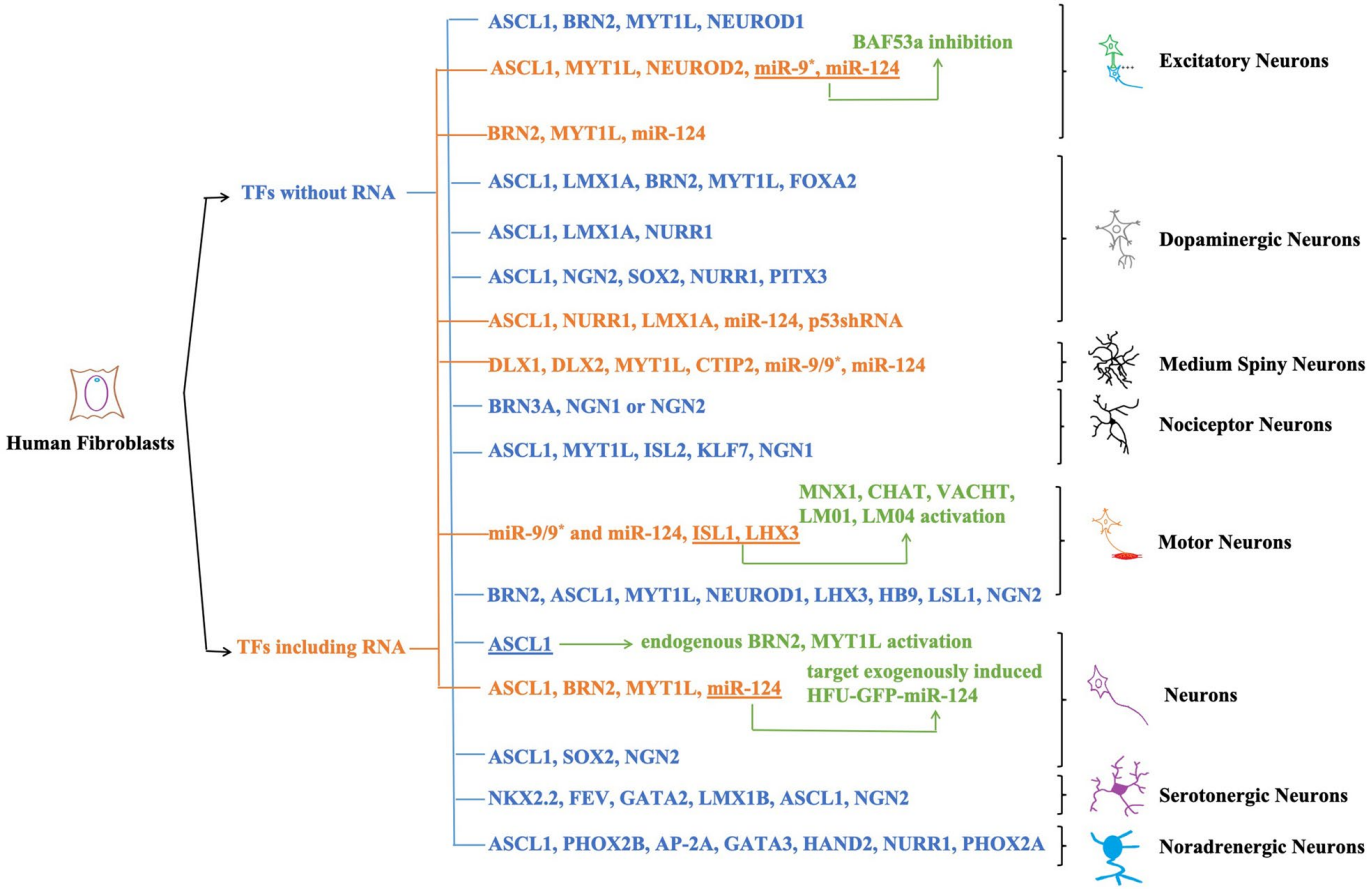
All the researches on reprogramming human fibroblasts into neurons by TFs since 2011. We divide them into two categories according to whether miRNA is involved in these researches (blue means participating while orange means the opposite). At the same time, we also summarize the mechanism of TFs reported in the corresponding article (green) and neuron types

Reprogramming is the trend of Modern Regenerative Medicine Research [39]. Direct reprogramming of human fibroblasts skips the pluripotency stage and avoids ethical problems as well as tumorigenicity caused by iPSCs. This article summarizes all studies on the reprogramming of human fibroblasts into neurons using TFs from 2011 up to now (Table 1). At the same time, according to the miRNA participating or not, we divide the studies into two categories and summarize the existing mechanisms and neuron types (Fig. 1). Earlier, the researchers have converted the mouse fibroblasts into neurons. Pang et al. first applied the idea to human fibroblasts, successfully reprogrammed them into neurons by using four TFs BRN, ASCL1, MYT1L, and NEUROD1 [19] and created a new chapter for the development of modern medical science. Subsequently, some scholars reprogrammed human fibroblasts into specific neurons, providing a theoretical basis for the production of specific subtypes of neurons from human cells [40, 41]. Yoo et al. first realized the direct transformation of fibroblasts into neurons by combining the determined factors with microRNA such as miR-9/9* and miR-124 (miR-9/9*-124) leading the study to a new chapter. It is considered that miR-9/9*-124 can interact with each other to work on the independent site of 3′ untranslation region on *BAF53a*, by which they can activate *BAF53b* or inhibit *BAF53a*. It proved they played a guiding role in the determination of neural fate and speculated that the combination of miR-9/9*-124 and different neurogenic TFs may induce different types of neurons. However, the induction process was still very complex and took months while it had a low success rate [42]. TH^+^ neurons were obtained by continuously expressing three TFs of Ascl1, Lmx1a and Nurr1 for six days, greatly shortened the experimental period [43]. Based on improving the culture conditions, human fibroblasts were reprogrammed into neurons by using only a single transcription factor ASCL1 for the first time, which could activate endogenous *MYT1L* as well as *BRN2* and simplify the experimental steps. However, the maturity of target cells obtained by this method is deficient [44]. However, the lentiviral vectors themselves may have many shortcomings such as possible the introduction of reverse transcription elements [45, 46] and Doxycycline regulatory system not suitable for clinical application (it contains elements from bacteria, which need to continuously transport doxycycline to keep genes active) [47]. To avoid these problems, miR-124 was combined with integrated enzyme deletion vectors, then inserted the combination into the four complementary binding sites, so that mRNA in fibroblasts which did not express miR-124 was not inhibited or degraded. When the transformation reached a stable neuronal fate, human neurons initiated the intrinsic miR-124, and then miR-124 binds to the miR-target sequence in vector-derived mRNA. In this way, they effectively inhibited the expression of TFs, realized the self-regulation of unintegrated transformation vectors (using microRNAs to shut down the reprogrammed gene only in cells that have reached the stable fate of neurons), and avoided the application of doxycycline and produced functional neurons [48]. However, the introduction of ectopic genes is not completely controllable, which limits its application in clinical application and so on. At the same time, there are some disadvantages such as complicated operation, long time consumption, and low induction efficiency, all of which weaken its practical application value.

**Table 1:** Reprogram human fibroblasts into neurons using transcription factors since 2011

Target cell type	Reprogramming factors	Effects on neural cells induction	References
Excitatory neurons	BRN, ASCL1, MYT1L	Convert human fibroblasts into functional neurons	Pang et al. 2011 [19]
	NEUROD1	Improve the efficiency of reprogramming human fibroblasts into TUJ1 positive neurons	
Excitatory neurons	ASCL1, MYT1L, NEUROD2	Improve the maturity of neurons which reprogram from human fibroblasts	Yoo et.al. 2011 [42]
		Improve the maturity of neurons which reprogram from human fibroblasts	
		Improve the maturity of neurons which reprogram from human fibroblasts	
	miR-9*	Induce the transformation of human fibroblasts into neurons	
	miR-124	Induce the transformation of human fibroblasts into neurons	
Excitatory neurons	BRN2	Unknown	Ambasudhan et al. 2011 [89]
	MYTL1	Unknown	
	miR-124	Regulate the activity of major antineuronal differentiation factors in the central system;inhibit non-neuronal genes in post-transcriptional neurons	
Dopaminergic neurons	ASCL1	Convert human fibroblasts into neurons	Pfisterer et al. 2011 [40]
	LMX1A	Promote conversion of neurons from human fibroblasts into dopaminergic neurons	
	BRN2	Convert human fibroblasts into neurons	
	MYT1L	Convert human fibroblasts into neurons	
	FOXA2	Promote conversion of neurons from human fibroblasts into dopaminergic neurons	
Dopaminergic neurons	ASCL1	Reprogram fibroblasts into TH^+^ neurons by combining with Nurr1	Caiazzo et al. 2011 [43]
	LMX1A	Increase the efficiency of fibroblasts reprogramming into TH^+^ neurons by cooperating with Ascl1 and Nurr1	
	NURR1	The vital determinant of the specification and survival of dopaminergic neurons in development and adulthood	
Motor neurons	BRN2, ASCL1, MYT1L, NEUROD1, LHX3	Instruct the formation of motor neurons during development	Son et al. 2011 [41]
	HB9, LSL1, NGN2	Improve the efficiency of reprogramming human fibroblasts into induced motor neurons (iMN)	
Dopaminergic neurons	ASCL1	Neuronal determination function; promote the generation of mDA neurons by cooperating with Nurr1 and Ngn2 during midbrain development; promote the maturation of mDA neurons	Liu et al. 2012 [21]
	NGN2	Neuronal determination function; a necessary factor for mDA neuronal development	
	SOX2	A hallmark of nervous system, start with the development of the nervous system in selected brain regions and the maintenance of neurons	
	NURR1	Increase maturation of DA neurons reprogrammed by human fibroblasts	
	PITX3	Increase maturation of DA neurons reprogrammed by human fibroblasts	
Neurons	ASCL1	Reprogram human fibroblasts into neurons	Chanda et al. 2014 [44]
Medium spiny neurons	DLX1, DLX2	miR-9/9*-124 combining with DLX1 and DLX2 is vital important to MSN's terminal differentiation (Mutations of the homeobox genes *DLX-1* and *DLX-2* disrupt the striatal subventricular zone and differentiation of late born striatal neurons.)	Victor et al. 2014 [98]
	MYT1L	Increase the number of MAP2 positive cells obtained by human fibroblasts reprogramming	
	CTIP2	Inhibit apoptosis of hematopoietic progenitor cells by overexpression	
	miR-9/9*	Control the assembly of neuron-specific ATP-dependent chromatin remodeling complexes during neural development; regulate the expression of anti-nerve genes	
	miR-124	Control the assembly of neuron-specific ATP-dependent chromatin remodeling complexes during neural development; regulate the expression of anti-nerve genes	
Neurons	ASCL1, BRN2, MYT1L	Unknown	Lau et al. 2014 [48]
	miR-124	Turn off the reprogramming gene expression of stable neurons by regulating the reprogramming gene; promote neurogenesis and regulate the activity of neurons	
Nociceptor, mechanoreceptor, proprioceptor neurons	BRN3A	A necessary factor for the differentiation of sensory neurons	Blanchard et al. 2015 [99]
	NGN1 or NGN2	A necessary factor for the differentiation of sensory neurons; the precursors of sensory cells express NGN1 or NGN2; NGN1 and NGN2 may be transactivated or have overlapping/equivalent activities during the reprogramming of human fibroblasts into sensory neurons	
Nociceptor neurons	ASCL1, MYT1L	Promote human fibroblasts reprogramming into different subtypes of neurons	Wainger et al. 2015 [93]
	ISL2	Effect is currently unclear, but the expression in situ shows more pain receptor specificity	
	KLF7	Maintain the expression of TRKA, promoting human fibroblasts reprogramming into nociceptors	
	NGN1	A necessary factor for the formation of nociceptor precursor expressing NTRK1 and postnatal nociceptors expressing TRPV1	
Dopaminergic neurons	ASCL1	Convert embryonic carcinoma cells into neurons, and lead to a rapid withdrawal of the cell cycle, possibly by inducing the cycle-dependent kinase inhibition P27KIP1	Jiang et al. 2015 [90]
	NURR1	Unknown	
	LMX1A	Unknown	
	miR-124	Significantly improve the efficiency of ANL (ASCL1, NURR1 and LMX1A) to generate TH^+^ cells; enhance the morphology of iDA neurons; increase the reprogramming efficiency of human fibroblasts into neurons	
	p53 shRNA	Promote fibroblasts transformation into iDA neurons	
Neurons (GABAergic and glutamate-energy neurons)	ASCL1, SOX2	Reprogram human fibroblasts into neurons	Zhao et al. 2015 [100]
	NGN2	Guide progenitor cells differentiating to neurons during development; improve the reprogramming efficiency of human fibroblasts into neurons	
Serotonergic neurons	NKX2.2, FEV, GATA2, LMX1B	Associated with serotonergic differentiation; be vital important for the specification and maturation of serotonergic neurons in the rodent midbrain dorsal raphe nuclei	Vadodaria et al. 2016 [94]
	ASCL1	Pro-neuronal transcription factors; be vital important for the specification and maturation of serotonergic neurons in the rodent midbrain dorsal raphe nuclei	
	NGN2	Pro-neuronal transcription factors	
Motor neurons	ISL1, LHX	Reprogram human fibroblasts into MAP2, TUBB3 and NCAM positive cells with complex neuronal morphology by the co-expression of LHX3 and ISL1 with miR-9/9 *-124	Abernathy et al. 2017 [91]
	miR-9/9* and miR-124	Trigger chromatin accessibility, DNA methylation, and reconfiguration of mRNA expression to induce the default neuronal state, but do not activate subtype-specific programs	
Noradrenergic neurons	ASCL1	Convert midbrain astrocytes into functional neurons	Li et al. 2019 [95]
	PHOX2B	Induce noradrenergic neuronal phenotypes; key factor for noradrenergic neurons’ generation	
	AP-2Α	Key factor for noradrenergic neurons’ generation	
	GATA3	GATA3 co-operating with Hand2	
	HAND2	Increases the level of noradrenaline released; key factors for noradrenergic neurons’ generation	
	NURR1	Promote the expression of mCherry and significantly increase the level of noradrenaline released; key factor for noradrenergic neurons’ generation	
	PHOX2A	Key factor for noradrenergic neurons’ generation

**Fig. 2:**
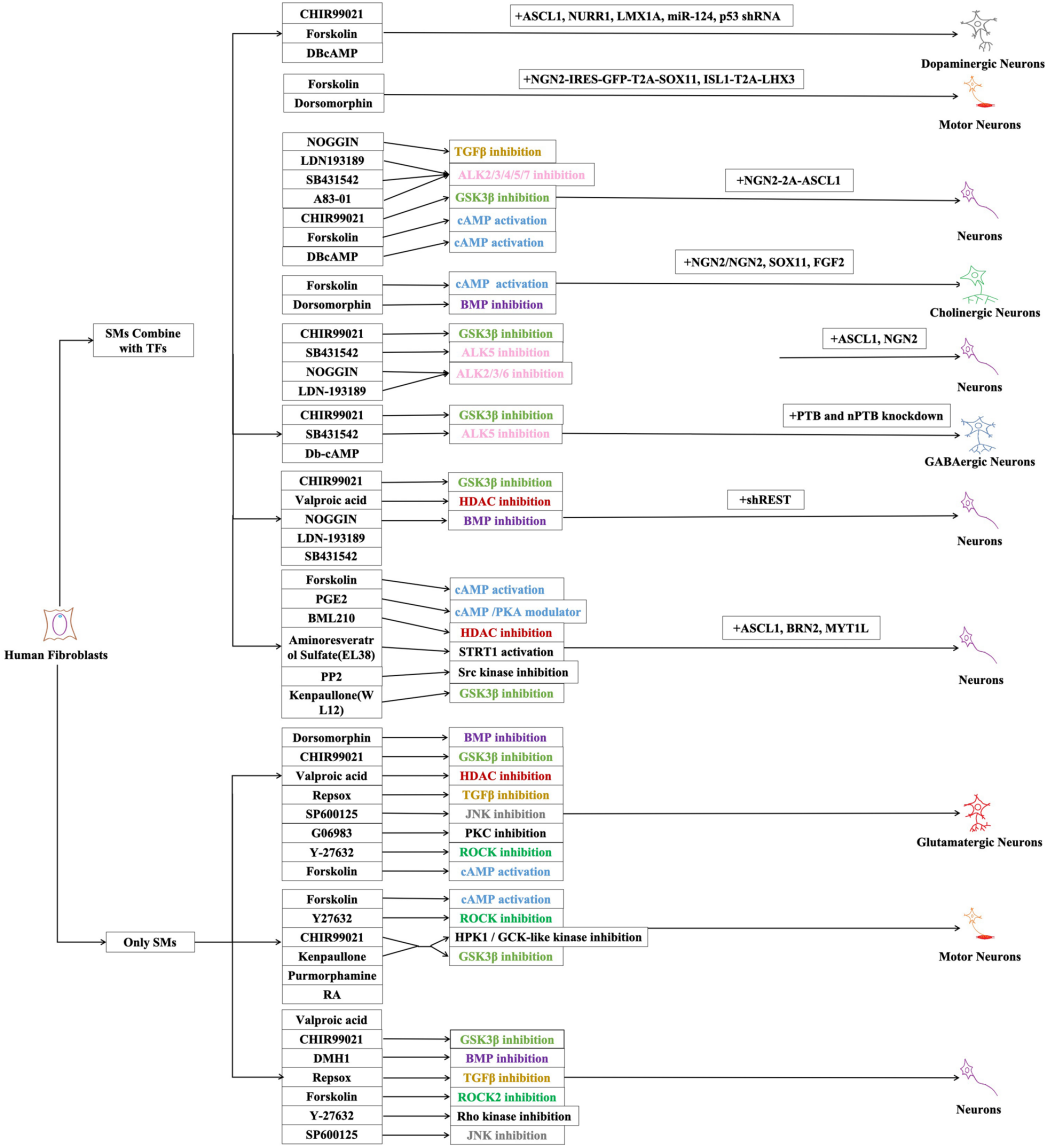
All the researches that SMs are applied to reprogramming human fibroblasts into neurons at present. According to whether TFs are involved in the reprogramming process of SMs, we classify them into two categories, and mark out the mechanism involved and the type of neurons obtained (the same mechanism of SMs uses the same color)

These years, a new way was found to reprogram fibroblasts through SMs. The combination of seven SMs including Forskolin, 2-methyl-5-hydroxytryptamine, D4476, Valproic acid, CHIR99021, 616,452, and Tranylcypromine could acquire iPSCs [49], and this method completely replaced the combination of four SMs found by Yamanaka. Compared with introducing TFs, SMs can replace exogenous genes and have succeeded in inducing cell transformation only by themselves. At the same time, SMs can permeate into the cells and have reversible biology activity [8]. Besides, SMs have many other advantages in application. For instance, they are easy to be operated while the time to deal with them can be controlled easily. What is more, the price of them is lower compared with TFs and can adjust combination and concentration by researchers [22]. The studies about human fibroblasts reprogramming by SMs have been reported since 2012 (Table 2), and they can be divided into two kinds depending on whether or not TFs take part in the reprogramming by SMs while the mechanisms and the type of neurons obtained are labeled (Fig. 2). At the very beginning, SMs were tried to combine with least TFs to reprogram human fibroblasts into neutral cells by using SB-431542 which inhibits ALK-5 or CHIR99021 which inhibits GSK3β together with NOGGIN and TFs ASCL1 and NGN2 to make cells have a higher positive rate in TUJ1 + and acquire a series of neurons in which about 20% are GABAergic neurons, 35% are glutamatergic neurons, 5% are 5-HT neurons and a bit tyrosine hydroxylase positive neurons. Besides, inhibiting the TGFβ-SMAD signal path can also accelerate reprogramming, which greatly improves the output and purity of neurons [20] while remaining disadvantages including complex operation and long-time cost. In addition, SMs such as Forskolin which activates cAMP signal conduction and Dorsomorphin which inhibits BMP signal conduction together with TFs NGN2, SOX11, FGF2 were used to reprogram human fibroblasts from embryos, new-born and adults, and neurons with characters of cholinergic neurons were got, most of which expressing CHAT and HB9 [50]. The advantage of ASCL1 and NGN2, both of which can decide the different distributions in the brain and spinal cord [51, 52] was taken to acquire mixed hypotype neurons [20]. At the same time, NGN was used to acquire single cholinergic neurons [50], suggesting that TFs are somehow of deciding effects on the hypotype of neurons. In 2016, RNA program mediated by PTB was successfully used to reprogram mouse fibroblasts into functional neurons, and it together with SMs such as CHIR99021, SB431542, Db-cAMP could reprogram fibroblasts into neurons, then the PTB path was explored during the reprogramming process instead of exploring the function of SMs and found two related control circles including PTB-REST-miR-124 circle and nPTB-BRN2-miR-9 circle which can accelerate the maturity of neurons. Since the failure of the reprogramming that uses shPTB alone, they choose to use shPTB and shnPTB in order in which they detected the expression of BRN2 and proved the importance of it in NPCs differentiation while the mechanism of how nPTB activate BRN2 remained further study [53]. Since 2015, scientists have tried to use SMs alone or gradually decrease kinds used in reprogramming to further simplify the operational steps which shorten the experiment cycle (3–5 days) and increase controllability [22, 54]. Eight SMs VCRFSGY (Valproic acid, CHIR99021, Repsox, Forskolin, SP600125, G06983, Y-27632) were used to transform human fibroblasts gradually into glutamatergic motor neurons [22]. In addition, six SMs (WL12, PGE2, Forskolin, BML 210, EL38, PP2) were used to reprogram human fibroblasts. During the process of filtrating, they found that the combination of inhibiting GSK3β and activating cAMP signal conduction can obviously increase the efficiency of reprogramming. When these two were used together with other molecules such as HDAC inhibitor, SRC kinase inhibitor, or SIRT1 activator, the purity of neurons would increase. And through increasing cAMP in cells mediated by Forskolin, GSK3β inhibitor, and ALK-2,3 and 5 inhibitions, neurons reprogramming could be promoted [55]. Then, five molecules including Kenpaullone (which turned to use CHIR99021 later), Forskolin, Y27632, purmorphamine, and RA were used to reprogramming human fibroblasts into motor neurons [8]. With the decrease in the kinds of SMs used and the clarity of the mechanism, especially being combined with gene editing which can achieve disease characteristic conveniently, it has been gradually applied to the establishment of disease models and is expected to be used in clinical [56].

**Table 2:** Reprogramming human fibroblasts into neurons using small molecules since 2012

Target Cell Type	Morphogens or Small molecules	Reprogramming Mechanism	Effects on neural cells induction	Reprogramming factors	Effects on neural cells induction	References
Neurons	CHIR99021	GSK3β inhibition	Suit the branch of neurons’ and axons’ growth	ASCL1, NGN2	Improve positive rate of Tuj1^+^ cells	Ladewig et al. 2012 [20]
	SB431542	ALK5 inhibition (Activin/Nodal/TGFβ pathway inhibition)	Unknown			
	NOGGIN, LDN-193189	ALK2/3/6 inhibition (BMP/SMAD signaling inhibition	Unknown			
Cholinergic neurons	Forskolin	cAMP activation	Induce massive production of Tuj1^+^ cells, which have neuron-like morphology	NGN2/NGN^2+^	Activate a cohesive pathway that determines a more homogenous neuronal subtype (cholinergic neuron)	Liu et al. 2013 [50]
	Dorsomorphin	BMP inhibition	Promote neuron survival and maturation cooperating with FSK, while DM itself does not affect neuron transformation	SOX11^+^	Promote the survival and maturation of neurons but does not specify the neuron subtype	
				FGF2	Improve reprogramming efficiency significantly	
Neurons	NOGGIN	TGFβ inhibition	Unknown	NGN2-2A-ASCL1	Unknown	Mertens et al. 2015 [101]
	LDN193189, SB431542, A83-01	ALK2/3/4/5/7 inhibition				
	CHIR99021,	GSK3β inhibition,				
	Forskolin, DBcAMP	cAMP activation				
Glutamatergic neurons	Valproic acid	HDAC inhibition	Activate cells through epigenetic modification	–	–	Hu et al. 2015 [22]
	CHIR99021	GSK3β inhibition	Promote the transformation of human fibroblasts to neurons and induce dopamine neurons to human ESCs			
	Repsox	TGFβ inhibition	Improve neuron survival			
	Forskolin	cAMP activation	Enable Ngn2 to convert human fibroblasts into cholinergic neurons			
	SP600125	JNK inhibition	Facilitate the neural reprogramming of AHDF transduced with OCT4 alone			
	G06983	PKC inhibition	Improve human pluripotent stem cells and induces neuritogenesis of Neuro-2a cells			
	Y-27632	ROCK inhibition	Maintain the viability of pluripotent stem cells and neurons			
	Dorsomorphin	BMP inhibition	Promote the survival and maturation of neurons			
Motor neurons	Forskolin Dorsomorphin	Unknown	All increase the population of Tuj1^+^ cells reprogramming from fibroblasts	NGN2-IRES-GFP-T2A-SOX11, ISL1-T2A-LHX3	Unknown	Liu et al. 2016 [92]
GABAergic Neurons	CHIR99021, SB431542, Db-cAMP	Unknown	Improve the efficiency of neuron differentiation	Order PTB knockdown and nPTB knockdown	Induce the transcription of miR-9, the activation of miR-9 made the induced neuron-like cells progress to functional neurons	Xue et al. 2016 [53]
				PTB knockdown and BRN2 expression	Facilitate the expression of MAP2 in neurons and promote the influx of Ca^2+^	
Neurons	Kenpaullone (WL12)	GSK3β inhibition	Increase reprogramming efficiency	ASCL1, BRN2, MYT1L	Unknown	Pfisterer et al. 2016 [55]
	PGE2	cAMP/PKA modulator	Increase reprogramming efficiency			
	Forskolin	cAMP activation	Unknown			
	BML 210	HDAC inhibition	Facilitate the recovery of pluripotency in reprogramming			
	Aminoresveratrol Sulfate (EL38)	SIRT1 activation	Unknown			
	PP2	Src kinase inhibition	Skip the SOX2 pathway, activate sirtuins complement and promote Klf4 expression			
Neurons	CHIR99021, SB431542, NOGGIN, LDN193189, Valproic acid	Unknown	Unknown	ASCL1, BRN2	Unknown	Drouin-Ouellet et al. 2017 [102] Shrigley et al. 2018 [103] Marina et al. 2020 [56]
				SHREST	miR-9 activation, miR-124 activation	
Motor neurons	Kenpaullone	GSK3β inhibition; HPK1 / GCK-like kinase inhibition	Enhance the binding of other small molecules to its target	–	–	Qin et al. 2018 [7]
	Later change to CHIR99021	cAMP activation	Promote the growth of neurons, which is weaker than that of Kenpaullone			
	Forskolin	ROCK inhibition	Unknown			
	Y27632	Unknown	Unknown			
	Purmorphamine	Unknown	Unknown			
	RA	Unknown	Improve the differentiation from pluripotent stem cells to motor neurons through the activation of RA and SHH signals			
Neurons	Valproic acid	Unknown	Unknown	–	–	Wan et al. 2018 [23]
	CHIR99021	Glycogen synthase kinase-3β inhibition	Improve the conversion of human fibroblasts to neurons			
	DMH1	BMP inhibition	Improve the conversion of human fibroblasts to neurons			
	Repsox	Transforming growth factor-β inhibition	Improve the conversion of human fibroblasts to neurons			
	Forskolin	ROCK2 inhibition	Improve migration of neural crest cells; enable neurogenin 2 to convert human fibroblasts into cholinergic neurons			
	Y-27632	Rho kinase inhibition	Facilitate the maintenance of pluripotent stem cells and neuron survival			
	SP600125	JNK inhibition	Enhance the neuronal conversion of fibroblasts			
Neurons	CHIR99021	GSK3 inhibition	Unknown	ASCL1, BRN2, ShREST	Unknown	Villanueva-Paz et al. 2019 [104]
	Noggin	BMP inhibition	Unknown			
	Valproic acid	HADC inhibition	Unknown			
	SB431542, LDN193189	Unknown	A neural fate inducing factor			
Dopaminergic neurons	CHIR99021, Purmorphamine, Y27632	Unknown	Unknown	ASCL1, NURR1, LMX1A, miR-124, p53 shRNA	Unknown	Jiali et al. 2020 [105]

## Reprogramming human fibroblasts into NSCs

**Fig. 3:**
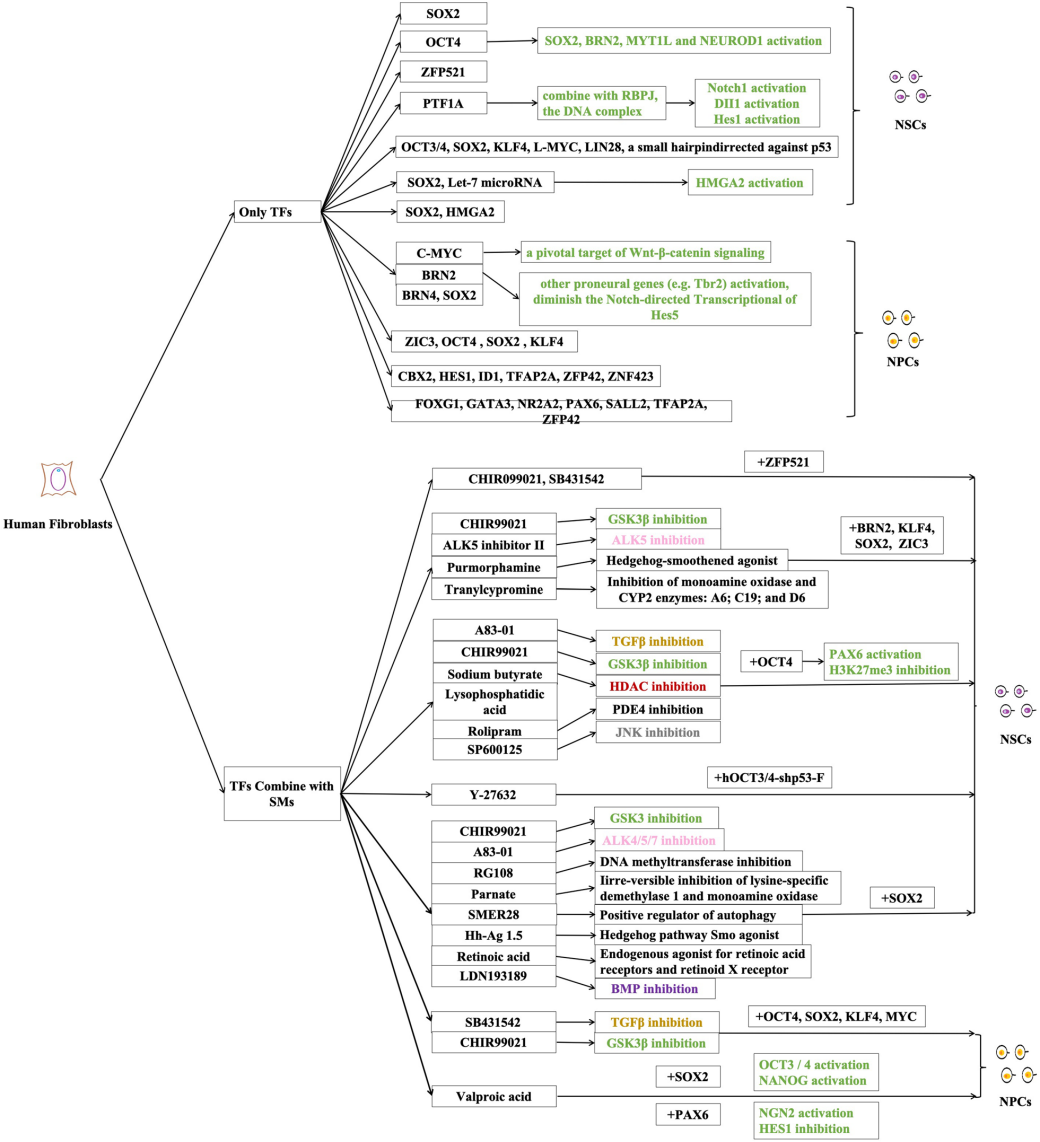
All the current researches on reprogramming human fibroblasts into NSCs by using TFs or TFs combining SMs. We divide them into two categories according to the different media used in these researches. They are only TFs and TFs combining SMs. At the same time, we also mark the mechanisms of TFs with green and the same mechanism of SMs uses the same color as Fig. 2

However, even if neurons can be obtained, due to their limited potential in proliferation and heterogeneity, it can be quite difficult to use them in transplantation and clinical trials, which may incur some limitations. At the same time, NSCs can proliferate and are able to transform into several kinds to acquire a large amount of relatively safe and homogeneous cell groups [57]. The patient-derived pluripotent NSCs can bypass the disadvantages of iPSCs and induced neural cells. NSCs have been proved to be a safe cell resource with a high survival rate, especially without tumor tendency [58, 59], which is a promising therapeutic strategy in regenerative medicine. Therefore, scientists deliberated whether human fibroblasts can be reprogrammed into proliferate neural stem/progenitor cells, which could become a potential infinite source of neurons and other types of neural cells. Table 3 in this passage sums up all the current studies of reprogramming human fibroblast cells into NSCs using TFs and Table 4 in this passage sums up all the current studies of reprogramming human fibroblast cells into NSCs using TFs together with SMs, they are divided them into two kinds by whether they used only TFs or TFs together with SMs to mark the current studies (Fig. 3). Five TFs including BRN4, SOX2, KLF4, C-MYC, E47 or four TFs including BRN4, SOX2, KLF4, C-MYC were used to reprogram mouse fibroblasts into NSCs, successfully differentiating them into all neural lineages not only in vitro but in vivo and determining its origin of ventral posterior brain by gene region analysis [60], laying the foundation for the transplantation location for reprogrammed NSCs. Noticing that SOX2 can maintain the proliferation of NSCs and inhibit the differentiation [61], people tried to reprogram fibroblasts into NSCs by using SOX2 alone or in conjunction with other TFs. SOX2 alone reprogrammed mouse and fetus fibroblast cells into NSCs and transplanted reprogrammed NSCs into the mouse brain without producing tumor, greatly excluding the defect of the teratoma formed from iPSCs, which proved that self-renewing NSCs with little tumorigenic potential can be acquired from reprogramming fibroblasts [62]. However, OCT4 successfully reprogrammed adult fibroblasts into NSCs in the experiment, it was found that SOX2 co-expressed with OCT4, but SOX2 were unable to complete the reprogramming alone. So, SOX2 was suggested to be downstream of OCT4, which means that the role of OCT4 is essential in adult fibroblast reprogramming [63]. Since then, the application of let-7 microRNA-targeted HMGA2 had significantly increased the efficiency of SOX2-induced reprogramming of human fibroblasts to human NSCs and greatly shortened the time needed [57]. SOX2 and HMGA2 were applied to reprogram fibroblasts from Niemann-Pick disease type C patients into NSCs which had self-renewal capacity with disease characters and expansion in vitro. Clinical drug treatments of the cell line have been proved effective, indicating that the reprogrammed NSCs line can provide a sufficient number of patient-specific cells for various therapeutic studies including drug screening, toxicity testing and even cell transplantation [64]. A single neurogenic factor ZFP521 reprogrammed fetal fibroblasts directly into NSCs, but the reprogramming of adult fibroblasts still required the use of a small molecular mixture of CHIR099021, SB431542 and valproic acid in hypoxia. The reprogrammed NSCs were transplanted into the brain of newborn mice and remained the ability to differentiate into neurons and astrocytes after 4 weeks. When transplanted into adult rat brain, NSCs survived and migrated to adjacent brain tissue while still held the characters of neural precursor [65]. SOX2 and ZFP521 have been used most frequently in recent researches and SOX2 has been supposed as the key regulator for fibroblasts to be reprogrammed to NSCs [66]. However, because SOX2 and ZFP521 are in connection with several cancers, researchers try to find other ways to reprogram fibroblasts. Single non-neural progenitor transcription factor PTF1A was applied to directly reprogram human fibroblasts into NSCs with activity and functions. NSCs obtained in this way have potentials to differentiate into three kinds of cells including neurons, astrocytes and oligodendrocytes [67]. What’s more, these neurons can form spontaneous postsynaptic activities. Further studies found that the interactions between PTF1A and RBPJ are prerequisites for the reprogramming of PTF1A and directly affect the formation of the neural ball gotten from reprogramming while reprogramming using PTF1A had better efficiency compared with that using SOX2. PTF1A can also activate the Notch path to realize the self-renewing and maintenance of NSCs. At the same time, the successful differentiation of the reprogrammed NSCs transplanted into the mouse suggested that this method has a bright application prospect. This study, for the first time, proved that single non-neural progenitor transcription factor was able to transform somatic cells into NSCs and suspected the hypothesis that SOX2 is the main transcription factor for direct NSCs reprogramming broadening the studying path of reprogramming fibroblasts into NSCs through TFs [17]. At the same time, the researches of transcription factor binding SMs to reprogram human fibroblasts into NSCs or NPCs had also begun to appear (Table 4). For example, four TFs OCT4, SOX2, KLF4, MYC were used to bind SB431542, CHIR99021 SMs and leukemia inhibitory factor reprogrammed human fibroblasts into NPCs, the NPCs were dissociated them into single cells and cultured them in neural differentiation medium, after 60 days, TUJ1^+^ and MAP2^+^ neurons appeared [68].

**Table 3:** All studies used transcription factors to reprogram human fibroblasts into NSCs and NPCs

Target cell type	Reprogramming factors	Reprogramming mechanism	Effects on neural cells induction	References
NSCs	SOX2	Unknown	Inhibit differentiation of NSCs and maintain its proliferation	Ring et al. 2012 [34]
NPCs	ZIC3, OCT4, SOX2, KLF4	Unknown	Play important roles in the maintenance of ESCs and neuroprogenitor cells pluripotency	Kumar et al. 2012 [106]
NSCs	OCT4	SOX2 activation	Induce the transformation from fibroblasts to proliferating NSCs	Mitchell et al. 2014 [63]
		BRN2 activation		
		MYT1L activation		
		NEUROD1 activation		
NPCs	SOX2	Unknown	Play an essential role in the maintenance of both ES cells and NSCs and prevents cell differentiation	Zou et al. 2014 [83]
	C-MYC	A pivotal target of Wnt-β-catenin signaling	Improve proliferation and promote reprogramming, regulate the neuronal differentiation of NPCs and the expansion of basal progenitors	
	BRN2	Other proneural genes (e.g. Tbr2) activation, diminish the Notch-directed transcription of HES5	Promote neurogenesis	
	BRN4	Unknown	Elevate neuronal differentiation and maturation from NSCs	
NSCs	let-7 microRNA	HMGA2 activation	Increase SOX2 reprogramming efficiency	Yu et al. 2015 [57]
	SOX2	HMGA2 activation	Unknown	
NSCs	Zfp521	Unknown	Maintain the NSC identity of the medulla oblongata	Shahbazi et al. 2016 [65]
NSCs	OCT3/4, SOX2, KLF4, L-MYC, LIN28	Unknown	Unknown	Capetian et al. 2016 [107]
	A small hairpin directed against p53	Unknown	Enhance the reprogramming process	
NSCs	SOX2	Unknown	Unknown	Sung et al. 2017 [64]
	HMGA2	Unknown	Increase reprogramming efficiency	
NSCs	PTF1A	Bind with RBPJ to form trimer DNA complex	Activate the reprogramming process, make NSCs maintain self-renewal ability and NSC identity and prevent excessive neuronal differentiation of NSCs	Xiao et al. 2018 [16] Kangxin et al. 2020 [67]
NPCs	CBX2, HES1, ID1, TFAP2A, ZFP42, ZNF423	Unknown	Increase proliferation of NSCs decrease apoptosis, keep rostral identity	Hou et al. 2017 [108]
	FOXG1, GATA3, NR2A2, PAX6, SALL2, TFAP2A, ZFP42		Conducive to neuron lineage differentiation and keep caudal identity

**Table 4:** All studies used transcription factors and small molecules to reprogram human fibroblasts into NSCs and NPCs

Target cell type	Reprogramming factors	Reprogramming mechanism	Effects on neural cells induction	Morphogens or small molecules	Reprogramming mechanism	Effects on neural cells induction	References
NPCs	SOX2	OCT3/4 and NANOG activation	Play a key role in the early stages of neurogenesis and the process of reprogramming cells to a pluripotent state; associated with multipotent and unipotent stem cells	Valproic acid	Unknown	Unknown	Maucksch et al. 2012 [109]
	PAX6	Concentration-dependent NGN2 activation, HES1 inhibition	Play a key role in the early stages of neurogenesis; be essential for neural stem cell proliferation, multipotency and neurogenesis; involve neural lineage determination SOX2 and PAX6 work together can reprogram human fibroblasts into neural precursor cells				
NPCs	OCT4, SOX2, KLF4, MYC	Unknown	Unknown	SB431542	TGFβ inhibition	Unknown	Lu et.al. 2013 [68]
				CHIR99021	GSK3β inhibition	Render neural progenitors in the entire neuraxis in a state of self-renewal	
NSCs	OCT4,	PAX6 activation, H3K27me3 inhibition	Unknown	A83-01	TGFβ inhibition	Unknown	Zhu et.al. 2014 [110]
				CHIR99021	GSK3β inhibition		
				Sodium butyrate	HDAC inhibition		
				Lysophosphatidic acid	Unknown		
				Rolipram	PDE4 inhibition		
				SP600125	JNK inhibition		
NPCs	OCT4, SOX2,KLF4, C-MYC	Unknown	Unknown	CHIR99021, SB431542	Unknown	Unknown	Meyer et al. 2015 [111]
NSCs	ZFP521	Unknown	Unknown	CHIR99021, SB431542	Unknown	Unknown	Shahbazi et al. 2016 [65]
NSCs	hOCT3/4-shp53-F	Unknown	Unknown	Y-27632	Unknown	Unknown	Azmitia et.al. 2018 [112]
NSCs	BRN2, KLF4, SOX2, ZIC3	Unknown	Have the ability of NSCs population maintenance and positive regulation of NPCs proliferation, induce neural crest identity of brain development and head development and the regional identity of a dorsal anterior hindbrain fate	CHIR99021	GSK-3 inhibition	Unknown	Thier et al., 2019 [113]
				ALK5 inhibitor II	ALK5 inhibition		
				Purmorphamine	Hedgehog-smoothened agonist		
				Tranylcypromine	Inhibitior of monoamine oxidase and CYP2 enzymes: A6; C19; and D6		
NSCs	SOX2	Unknown	Unknown	CHIR99021	Potent and selective GSK-3 inhibition	Unknown	Yanying et al. 2020 [114]
				A83-01	Potent inhibition of TGF-β type I receptor ALK5 kinase, type I Activin/Nodal receptor ALK4 and type I nodal receptor ALK7		
				RG108	Non-nucleoside DNA methyltransferase inhibition		
				Parnate	Iirre-versible inhibition of lysine-specific demethylase 1 and monoamine oxidase		
				SMER28	Positive regulator of autophagy		
				Hh-Ag 1.5	Potent Hedgehog pathway Smo agonist		
				Retinoic acid	Endogenous agonist for retinoic acid receptors and retinoid X receptor		
				LDN193189	Cell permeable BMP signaling inhibition

It is believed that in the near future, there will be researches on reprogramming human fibroblasts into neurons using SMs alone. Therefore, the mechanism and necessary conditions of direct reprogramming human fibroblasts into NSCs still need to be further explored. Even though, the advantages of this method are still obvious. NSCs have the potential of self-renewal and differentiation. The advantages of this method are avoiding ethical problems and having the genetic characteristics of patients without immune rejection. Compared with iPSCs, the method can not only obtain neurons faster and more efficiently but also selectively avoid the use of oncogenes. But at the same time, there are still remaining some shortcomings. For example, the reprogramming rate of human fibroblasts to neurons is still lower than that of mouse fibroblasts. It is still unknown whether the neuron is a functional neuron suitable for the pathological environment and whether the induction process leads to the abnormal expression of other key neuron genes in neurons. Also, the risk of genomic mutation in cell reprogramming still exists [69]. At the same time, whether the cells obtained by in vitro reprogramming can survive in vivo and play their correct physiological functions is still a major research direction.

## Reprogramming of fibroblasts into neurons in vivo

There are also some scientists who envisage using in vivo reprogramming. This method has many advantages, such as avoiding transplantation damage, gene mutation in the process of culture, teratoma generation while benefiting from the micro-environment in vivo. But at the same time, there are many challenges. The safe and effective delivery method of reprogramming factors is still unknown. How to increase the efficiency of reprogramming, how to avoid the miss-distance effect of reprogramming and how to eliminate potential side effects are all the problems faced at present [69].

The core focus of regenerative medicine is to develop individual-specific cell therapies to treat injuries caused by aging, disease and trauma through regeneration of damaged tissues. At present, in vivo reprogramming is widely considered as one of the most promising technology for individual-specific cell therapy [70].

GFP-labeled fibroblasts were implanted into the striatum and hippocampus of mice with inactivated neurotransformation factors and then activated neurotransformation in vivo by drinking water containing doxycycline [71], proving that it is possible to reprogram fibroblasts into neurons in vivo. However, due to the low speed of in vivo reprogramming in this way, it is necessary to provide exogenous stimuli to promote its differentiation into neurons such as periodic biphasic pulse‐like currents generated by Triboelectric Nanogenerator [72] and electromagnetic force to promote the effective transformation of fibroblasts into neurons in vivo [73]. These two techniques effectively accelerate the efficiency of in vivo reprogramming.

Although in vivo the reprogramming process is initiated mainly by the ectopic expression of key TFs, the reprogramming process and the final cell type are also highly influenced by the microenvironment. Regional differences in different places of body (including extracellular matrix (ECM), cytokines, neurotransmitters, etc.) can significantly affect the results of in vivo reprogramming. For instance, in the adult striatum, a majority of the Sox2-neurons were Cr^+^ inhibitory neurons, whereas those in the adult spinal cord are Vglut2^+^ excitatory neurons, although all of these reprogrammed cells pass through an Ascl1^+^ progenitor stage. The striatum and neocortex also demonstrate a differential response to ectopic Neurog2 expression and growth factor treatments. Neurog2-neurons in the striatum exhibit properties of striatal projection neurons, whereas those in the neocortex are largely glutamatergic ones [74].

In vivo reprogramming has many advantages, such as avoiding the injury of transplantation, gene mutation and teratoma in the process of culture while benefiting from the microenvironment in vivo. Therefore, in vivo reprogramming has great prospects. However, it is considered that in vivo reprogramming still faces great challenges, mainly in the following four aspects: 1. Ensuring the survival of neurons after reprogramming in vivo; 2. Regeneration of specific subtypes of neurons; 3. Reconstruction of neural circuits after reprogramming in vivo; 4. Whether newly reprogrammed neurons can restore lost brain or spinal cord function caused by injury or disease [75].

So, in the face of so many challenges, can we find a safer and more effective way to make use of some of the advantages here, such as benefiting from the in vivo microenvironment? If we simulate in vivo microenvironment in vitro, we may be able to obtain better neurons through safer methods. Researches about the effect of extracellular microenvironment on the fate of stem cells are increasing. Extracellular microenvironment effecting on cells can be divided into three aspects. The first is biochemistry, including adhesion ligands, immobilized growth/morphological factors, ligand conformation and ligand/factor spatial patterns while the second is biochemistry and biophysics, including stereo ligand mode to regulate cell shape, RGD modulus regulation and force-induced protein unfolding to express hidden sites. At the same time, the third is biophysics, including ECM modulus, force/pressure, morphology and dimension. These factors influence their next signal transduction including nuclear shape/transport, biochemical signal transduction, chromatin remodeling, cytoskeleton remodeling/isolation of cytoplasmic matrix and mechanically sensitive ion channels. These signal transduction alters many cellular biological functions, such as cell lineage stereotyping, survival/ apoptosis, self-renewal/ proliferation and spatial localization [76]. It is a good choice to use one or several of these factors to promote or reprogram human fibroblasts directly into NSCs which is a less tried area and can be a safer approach.

## Application of physical factors in the reprogramming of fibroblasts into neurons

Among various factors of the extracellular microenvironment, biophysics has become a research hotspot in stem cell tissue engineering. The first thing that attracted people's attention was Engler et al. who proved that different modulus of elasticity of ECM leads to stem cells differentiating in different directions, including neurons, muscle cells and bone cells [77]. Manipulating the growth microenvironment of fibroblasts growth can also control the fate of cell growth. However, there are very few researches reprogramming human fibroblasts into neurons by physical factors alone. Radio frequency microcurrent generated by REAC directly reprogrammed human fibroblasts into neurons [78], which has been shown to alleviate symptoms in patients with Alzheimer's disease [24]. The radio frequency microcurrent generated by the interaction of weak electromagnetic fields (EMF) produced by REAC is used to induce transcription of tissue-restricted genes, such as *NEUROGENIN 1*, which promotes neurogenesis while OCT4, SOX2, C-MYC, NANOG and KLF4 increase in early transcription within 6–20 h and then decrease. The former increases the expression of *TUJ1* and promotes the differentiation of human fibroblasts into neurons, while the latter bypasses the continuous reprogramming of iPSCs-like state by REAC and uses physical stimulation to make human fibroblasts bypass iPSCs state and reprogram directly into neurons [78].

It was found that the transcriptional patterns of NSCs cultured in 3D were more different from those cultured in 2D-PDL and 2D-COL [79], and the expression levels of the stem genes *Rex-1, Sox2, Oct4, and Nanog* of MSCs on 3D-COL were higher than TCP, and they had higher colony-forming ability. It indicated that 3D culture could be used as a factor to maintain the pluripotency of stem cells, and pluripotency factors of these stem cells were also those reprogramming factors. Hence, for verifying whether three-dimensional culture could play a role in the reprogramming process, mouse embryonic fibroblasts (MEFs) were cultured in petri dishes and 3D-COL and found that the ES markers of MEFs in 3D-COL were highly expressed [80]. In the same year, Su, G et al. validated this hypothesis and reprogrammed it using only the physical factor of 3D culture. They cultured MEFs on a low-adherence petri dish to form spheres and found that the expression of NPC-related genes in spheres was up-regulated, which could proliferate and differentiate into neurons, astrocytes and oligodendrocytes with physiological functions [81]. After that, three more reports on reprogramming human fibroblasts into induced NPCs using 3D culture and then inducing neurons were reported. 3D culture accompanied with TAT-SOX2 protein transduction was applied to induce human fibroblasts into NPCs which can be differentiated into TUJ1^+^ cells both in vitro and in vivo [82]. And the combination of SMs and TAT-mediated protein transduction of SOX2 and LMX1A in a 3D sphere culture was used to directly convert human fibroblasts to induced dopaminergic neural progenitor-like cells which could be induced to dopaminergic neural cells [83]. There was also a study showing that we could succeed to obtain NPCs which can later be induced into TUJ1^+^ cells by changing physicochemical culture properties from monolayer culture into a suspension in the presence of a chemical DNA methyltransferase inhibitor agent, Azacytidine [84].

Because few experiments have been done to reprogram human fibroblasts into neurons by using physical factors alone, this paper also lists the application of physical factors binding with TFs in inducing fibroblasts to reprogram into neurons. Cell-terrain interaction can also affect the process of fibroblast-neuron reprogramming. Fibroblasts transfected with TFs Ascl1, Pitx3, Nurr1 and Lmx1a could be reprogrammed iDA neurons, and nanogroove substrates could enhance the process. Topological clues provided by nanotopography could not only induce differentiable cell arrays and elongation along the groove axis, but also improve conversion efficiency, effectively promoting directly reprogramming of MEFs into functional DA neurons. The signal generated by the nanoscale matrix can be transferred to the nucleus through the cytoskeleton, so cytoskeletal tissue can significantly amplify the forced expression of DA gene and subsequent MET activation, which may trigger significant epigenetic changes [85]. And grating morphology on the substrate could promote the efficiency and purity of transcription factor reprogramming [86], which showed the effect of substrate morphology on direct reprogramming. Fibroblasts transfected with ASCL1, MYT1L and BRN2 were cultured on a grating to increase the purity and transformation efficiency of neurons. Microarrays and subsequent qPCR analysis showed that the expression of BMP5 in neurons produced on the grating morphology decreased. It is known that blocking BMP pathway with SMs can not only promote the differentiation of human ESCs and iPSCs [87], but also improve the efficiency of human fibroblasts reprogramming into neurons [20]. At the same time, there was a significant decrease in the branching of the products on the grating plate. On the one hand, the expression of *SLIT3, ARTEMIN* and *NETRIN G* genes related to the axon branching in the products on the grating plate was down regulated, which reduced the axon of neurons. On the other hand, *BMP5*′s activation promotes the formation of dendritic spines in neurons, and its inhibition leads to the decrease of dendritic spines in neurons. The decrease of MAP2 in neurons can be observed [86]. In other reports, physical factors and TFs were used together to realize reprogramming. Electromagnetic force is the physical energy that occurs between charged objects in EMF. Currently, experiments have shown that electromagnetic energy can promote the effective transformation of fibroblasts into iDA neurons both in vitro and in vivo. AuNPs (nanoparticles) exposed to EMF promote the induction of histone acetyltransferase Brd2, which acetylates histones H3K27 and H4K12 and leads to the opening of chromatin. As a result, the gene expression of neurons is enhanced, and the efficiency of dopaminergic neuron reprogramming in vivo and in vitro is significantly improved [73]. The reprogramming of fibroblasts to neurons was simulated by stimulating microcurrent in vivo through bi-directional pulsed current generated by triboelectric nano-engine. Exposure of cells transfected with Brn2, Ascl1 and Myt1l to triboelectricity stimulator (TES) activates Ca^2+^ channels, increases Ca^2+^ inflow, and activates downstream ERK1/2 pathways and protein kinase C, resulting in increased phosphorylation of ERK1/2. This suggests that microcurrent directly reprogrammed fibroblasts into neurons by inducing improvements in a series of cell events [72]. Surprisingly, 3D ECM hydrogel simulating brain microenvironment could accelerate the MET process and have a low contractile force which prevents YAP from entering the nucleus. H3K4me3 and H3K27ac occurred in the promoter region of TUJ1, which activated the relevant transcription and promoted reprogramming directly into functional neurons [88], which further illustrated the importance of extracellular microenvironment for reprogramming.

**Fig. 4:**
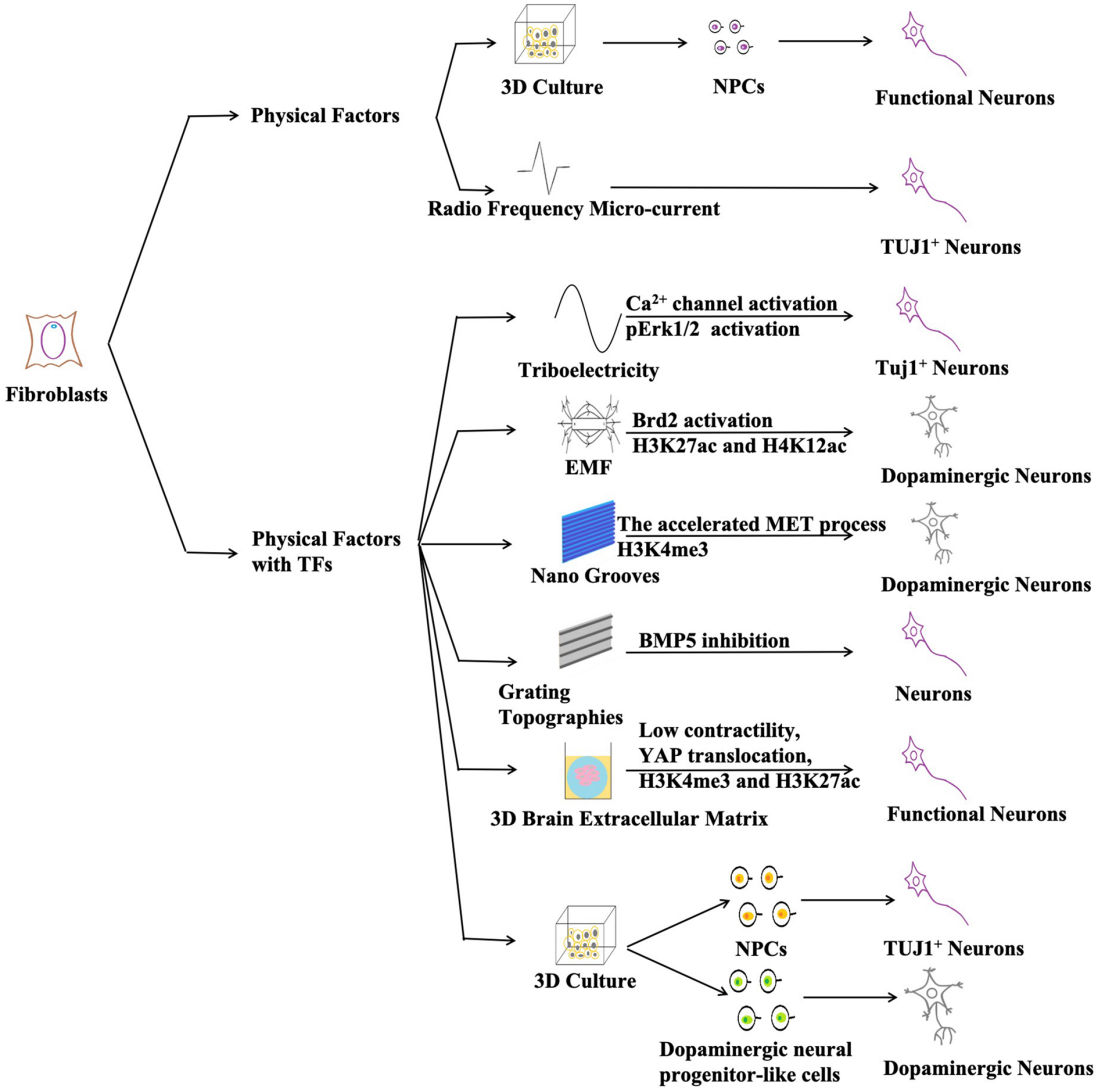
All studies of fibroblasts involved in reprogramming into neurons. We divide these studies into two categories according to whether physical factors combine with SMs, and mark the mechanism of the study and the types of neurons obtained

These studies are divided into two categories according to whether physical factors binding SMs or not and the mechanisms and types of neurons are also marked (Fig. 4). It is not difficult to see that there are few studies on reprogramming of physical factors, and more roles and mechanisms need to be studied.

## Outlook and concluding remarks

With the development of disease research and medicine, regenerative medicine has gradually become the focus of attention. In the process of acquiring neurons, the method of differentiation of iPSCs is the most classical, but some extreme problems such as tumorigenicity et al. can’t be avoided. Therefore, the direct transformation from somatic cells to neurons has become the necessity of scientific development and the target cells reprogrammed by human somatic cells work well in avoiding the body reaction, such as immune rejection and ethical problems, however, there is still a risk of genomic mutation in cell reprogramming itself, and we can avoid the use of carcinogenic factors in a variety of reprogramming methods. Among somatic cells, fibroblasts have a wide range of sources and are easy to obtain, which are the best choice.

Some research results of this paper clearly pointed out that the subtypes of neurons that had been directly reprogrammed into neurons through human fibroblasts, for example, excitatory and dopaminergic neurons were obtained by using TFs or TFs in combination with miRNA [19, 21, 40, 42, 43, 89, 90], dopaminergic neurons could also use TFs in combination with EMF or nano grooves [73, 85]; motor neurons could be obtained by TFs [41], TFs binding miRNA [91] or TFs binding SMs [8, 92]; nociceptor, serotonergic and noradrenergic neurons could be obtained by TFs [93–95]; cholinergic and GABAergic neurons could be obtained by TFs and SMs [50, 53]; glutamatergic neurons could be obtained through SMs [22]. The proposal of these neural subtype acquisition methods is helpful for the construction or expansion of the repertoire of brain cell subtypes.

**Fig. 5:**
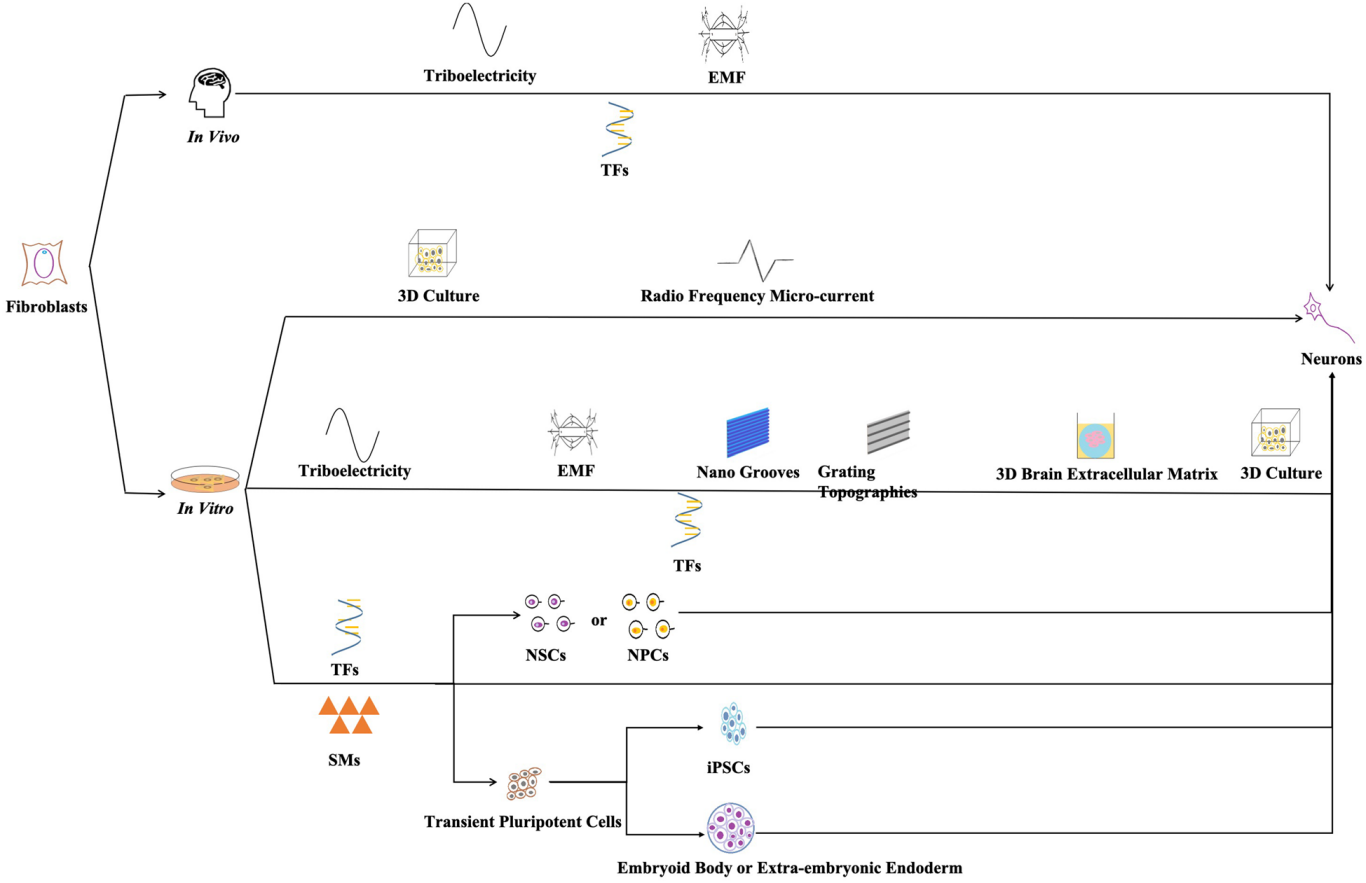
All current methods for directly reprogramming human fibroblasts into mature neurons. These methods had physical factors combined with TFs in vivo and physical factors, physical factors combined with TFs, TFs, SMs, and TFs combined with SMs passing in or out of intermediate stages in vitro. Because there were few related studies using physical factors, specific physical factors were also listed here

This paper summarizes all current methods for directly reprogramming human fibroblasts into mature neurons (Fig. 5). Firstly, human fibroblasts are reprogrammed by introducing TFs. This method is mature and diverse, different types of mature neurons can be obtained to meet the needs of different studies, but the blanks on its mechanisms still remain. At the same time, the introduction of TFs leads to the incomplete controllability of genes. Therefore, this method still has some risks and is not suitable for clinical application at present. Subsequently, the emergence of SMs has gradually replaced TFs. At the beginning, SMs needed to be combined with TFs to reprogram human fibroblasts into neurons, before long, they can be used independently to obtain neurons. SMs, with their advantages of safer and easier operation, have stepped onto the stage of regenerative medicine. It is still not so systematic and comprehensive that can be used in further researches.

The mature neurons obtained by human fibroblasts reprogramming have opened a new chapter for alternative therapy, but the problem of non-regeneration of neurons can’t be overcome. Therefore, if they are used in clinical therapy, they may only have a short effective time. When the cells were apoptotic, disease wound recurrence. On the contrary, reprogramming fibroblasts into NSCs can avoid this problem, prolong the effective time of treatment and improve the living standards of patients. The SMs involved in most studies of reprogramming human fibroblasts into neurons, NSCs and NPCs reported most of the mechanisms at present. According to Figs. 2 and 3 we have summarized the SMs mechanism, the same SMs mechanism uses the same color, it can be seen that these mechanisms, such as GSK3β inhibition, cAMP activation, ALK family inhibition, TGFβ inhibition and HDAC inhibition, are more important in this reprogramming process. These mechanisms have important research value in the studies of physical factors involved in neurons or NSCs reprogramming. However, the methods of reprogramming human fibroblasts into NSCs stagnated at the level of TFs, there was currently no study using SMs alone in this field, and the mechanism and necessary conditions of TFs were still unclear, so it had a very broad research prospect.

At the same time, in vivo reprogramming has attracted the attention of researchers because of its own advantages, which can avoid uncontrollable factors in vitro culture and injury in transplantation process. It is also found that the organism can provide a more stable and suitable microenvironment for cell transformation. However, it is also impossible to detect the degree of fibroblasts transformation and the quality of target cells in vivo, so there are certain risks which need further study. By simulating in vivo microenvironment in vitro, the risks of unknown factors on in vivo reprogramming can be effectively avoided. Considering the importance of physical factors in in vivo microenvironment, reprogramming of fibroblasts is greatly likely to be achieved by simulating the physical environment in vivo.

At present, researches related to physical factors are still scarce in the researches of reprogramming fibroblasts into neurons, as mentioned earlier, the Nano grooves accelerated the MET process, while brain extracellular matrix had low cytoskeleton organization and so on. Studies had confirmed that the initial MET process of mesoderm to ectoderm reprogramming was important [96], and cytoskeleton remodeling was conducive to the somatic reprogramming process [97], therefore, many physical factors can improve reprogramming efficiency, so they still have very strong application prospects. In short, the advantages of physical factors in the reprogramming of fibroblasts can be mainly concluded in the following points: 1. There is no need to apply TFs by viruses or other ways, so as to avoid a series of toxic and side effects of introduction methods on the body; 2. It does not involve direct manipulation of genetic materials, so as to avoid gene mutation and teratoma and other malignant phenomena in the induction process. Therefore, physical factor reprogramming has greater security and applicability, therefore it is more suitable for the establishment and experiment of disease models and clinical treatment of patients.

At present, the application methods of physical factor direct reprogramming mainly include two aspects. One is to use electromagnetic and electric fields which can directly affect body to carry out in vivo direct reprogramming, to treat patients through in vitro measures. This method can make use of the microenvironment in vivo and avoid the complex and risky implantation process, which greatly reduces the toxic and side effects on the body. However, due to the complex microenvironment in vivo, it is difficult to accurately control the number and type of neurons, so it has certain limitations. Another is the use of matrix-related physical factors, such as hardness, morphology and so on, to promote direct reprogramming in vitro. Although neurons obtained by reprogramming in vitro have problems of survival and exerting physiological functions in vivo, the treatment of clinical diseases through the combination of physical factors and SM compounds or TFs has a large room for development.

As far as the current research situation is concerned, the biggest problem physical factor reprogramming faces is how to achieve direct reprogramming of using simple physical factors. Except for this, the methods in this field are especially scarce and research in being is not deep enough. In the future, the application of physical factors individually to achieve neurons reprogramming may flourish, which will provide more hope and possibilities for the treatment of nervous system diseases.

## Abbreviations

iPSCs: Induced pluripotent stem cells
5-HT: 5-Tyrosine hydroxylase
CRT: Cell replacement therapy
ESCs: Embryonic stem cells
NSCs: Neural stem cells
TFs: Transcriptional factors
SMs: Small molecules
REAC: Radio electric asymmetric conveyer
NPCs: Neural precursor cells
COL: Collagen
iMN: Induced motor neurons
ECM: Extracellular matrix
MSCs: Mesenchymal stem cell
MEFs: Mouse embryonic fibroblasts
EMF: Electromagnetic fields
TES: Triboelectricity stimulator
MET: Mesenchymal-epithelial-transition
